# The burden of hyperkalemia in Germany – a real world evidence study assessing the treatment and costs of hyperkalemia

**DOI:** 10.1186/s12882-020-01942-2

**Published:** 2020-08-08

**Authors:** Jennifer Scarlet Haas, Kim-Sarah Krinke, Christopher Maas, Thomas Hardt, Isabella Barck, Sebastian Braun

**Affiliations:** 1 Xcenda GmbH, Lange Laube 31, 30159 Hannover, Germany; 2 Vifor Pharma Gruppe, Pharma Deutschland GmbH, Baierbrunner Str. 29, 81379 Munich, Germany

**Keywords:** Hyperkalemia, Burden of disease, Healthcare costs, Dialysis, Claims data analysis, Matching

## Abstract

**Background:**

Hyperkalemia (HK) can affect health outcomes and quality of life, as it is referred to as a potentially life-threatening condition caused by an increased serum potassium concentration in the blood. Patients suffering from heart failure or chronic kidney diseases are at a higher risk of HK, which can further be amplified by the treatment received. To date, data on HK prevalence is lacking for Germany and the aims of this study were to assess HK and compare health-relevant outcomes and healthcare costs between HK patients and non-HK patients.

**Methods:**

The InGef research database containing healthcare claims of over 4 million individuals in Germany was utilized for this retrospective, matched cohort analysis. Patients with non-acute outpatient treated and a subgroup of patients with chronic HK, were identified in 2015 with an individual 1 year pre- and post-index period, taking the first observable HK diagnosis/treatment in 2015 into account as the index event. To identify non-acute outpatient treated HK patients, at least two ICD-10-GM diagnosis codes E87.5 “Hyperkalemia” and/or prescriptions of polystyrene sulfonate were required. Chronic HK patients had additional diagnoses and/or prescriptions in all quarters following the first observable HK diagnosis. Patients without HK were matched 1:1 to the respective HK cohorts.

**Results:**

In the year 2015, 3333 patients with non-acute outpatient treated HK were identified of which 1693 were patients with chronic HK. After matching, 3191 and 1664 HK patients and controls were available for analysis. A significantly higher number of hospitalizations was observed for both HK cohorts in comparison to their matched controls. Dialysis initiation as well as the healthcare costs were higher for both HK cohorts when compared to their matched counterparts.

**Conclusions:**

The disease burden was higher for patients with HK, based on a higher proportion of patients with dialysis initiation and higher healthcare costs.

## Background

Hyperkalemia (HK) is a potentially life-threatening metabolic condition characterized by an increased serum concentration of potassium. Normal values of potassium have a range from 3.5 to 5 mmol/l, and hyperkalemia is defined by a value of > = 5.1 mmol/l [1, 2]; some authors prefer a low limit of 5.5 mmol/l [3, 4].

Clinical symptoms of hyperkalemia are inconstant and unspecific (fatigue, muscular weakness), however, hyperkalemia can cause arrhythmia and heart arrest without pre-occurring clinical signs [5]. The condition of hyperkalemia is usually detected in the context of routine laboratory screening. In the light of possibly life-threatening consequences, a finding of hyperkalemia implies an urgent demand for medical clarification and treatment [6].

The predominant risk factor of hyperkalemia is advanced chronic kidney disease (CKD). In patients with CKD ≥3 (GFR < 60 ml/min), the decreased ability to excrete potassium, especially in combination with diabetes mellitus and/or heart failure frequently results in recurrent and chronic hyperkalemia [7]. Medication (e.g. non-steroidal anti-inflammatory drugs (NSAID), renin-angiotensin-aldosterone system (RAAS)-Inhibitors and lifestyle (irregular nutrition and circadian rhythm) are additional risk factors [8, 9].

The true incidence and prevalence of hyperkalemia in the general population is not known, as there are no population-wide studies that examine it [10]. A retrospective analysis based on a selected Canadian population (≥ 65 years) demonstrates that 2.6% of the individuals from the emergency department and 3.5% of the hospitalized patients were hyperkalemic [11]. Further retrospective US researches deliver similar incidence rates between 2.5 and 3.2% [12, 13]. However, due to the low sensitivity, the true frequency in the general population is assumed to be considerably higher.

At present, little is known about the overall non-acute incidence and prevalence of HK in Germany and the related health resource utilization and costs caused by HK. Additionally, recent studies have focused on the occurrence of HK within a specific disease or within an inpatient setting, rather than on the general prevalence of HK independent of underlying diseases. The differentiation between acute and non-acute HK is important for medical and pharmacoeconomic reasons: Patients with acute HK receive treatment in emergency units following a standardized sequence of membrane stabilization, potassium shift and potassium elimination, usually hemodialysis [14], while patients with non-acute HK usually receive individualized therapy including dietary consulting, medication, and lifestyle adjustment [15]. Therefore, the aims of this claims database analysis were to assess the prevalence of outpatient treated HK and associated comorbidities as well as health-related resource use from the German healthcare perspective. Additionally, health-relevant outcomes as well as healthcare costs between (a) non-acute outpatient treated HK patients and non-HK patients as well as (b) chronic HK patients and non-HK patients in the German Statutory Health System (SHI) were compared.

## Methods

The goal of this analysis was to assess non-acute outpatient treated HK patients and chronic HK patients in terms of health-relevant outcomes. Further, the occurrence of hospitalizations, healthcare costs as well as dialysis initiation in relation to non-HK patients was explored. A retrospective design was applied using claims from the “Institut für angewandte Gesundheitsforschung Berlin” (InGef) research database. The InGef database consists of 6.7 million covered lives from about 60 different health insurances and includes the healthcare resource utilization and costs of services in an anonymized case-by-case individual format. For scientific research projects, an adjusted analysis sample of the InGef database has been drawn, including approximately 4 million covered lives which represent the German population in terms of age and gender (representing 4.8% of the German population and 5.6% of the German SHI population). Further, the analysis sample has been proven to have good external validity in terms of morbidity, mortality and drug use [16].

### Patient identification

The complete study period included data from January 1st, 2014 through December 31st, 2016 with an enrollment period from January 1st, 2015 through December 31st, 2015. Selected patients were required to be observable within an individual pre-index period of 4 quarters and an individual post-index period including the index quarter and 3 consecutive quarters. The first observable diagnosis or prescription marked the index event/quarter.

**Fig. 1:**
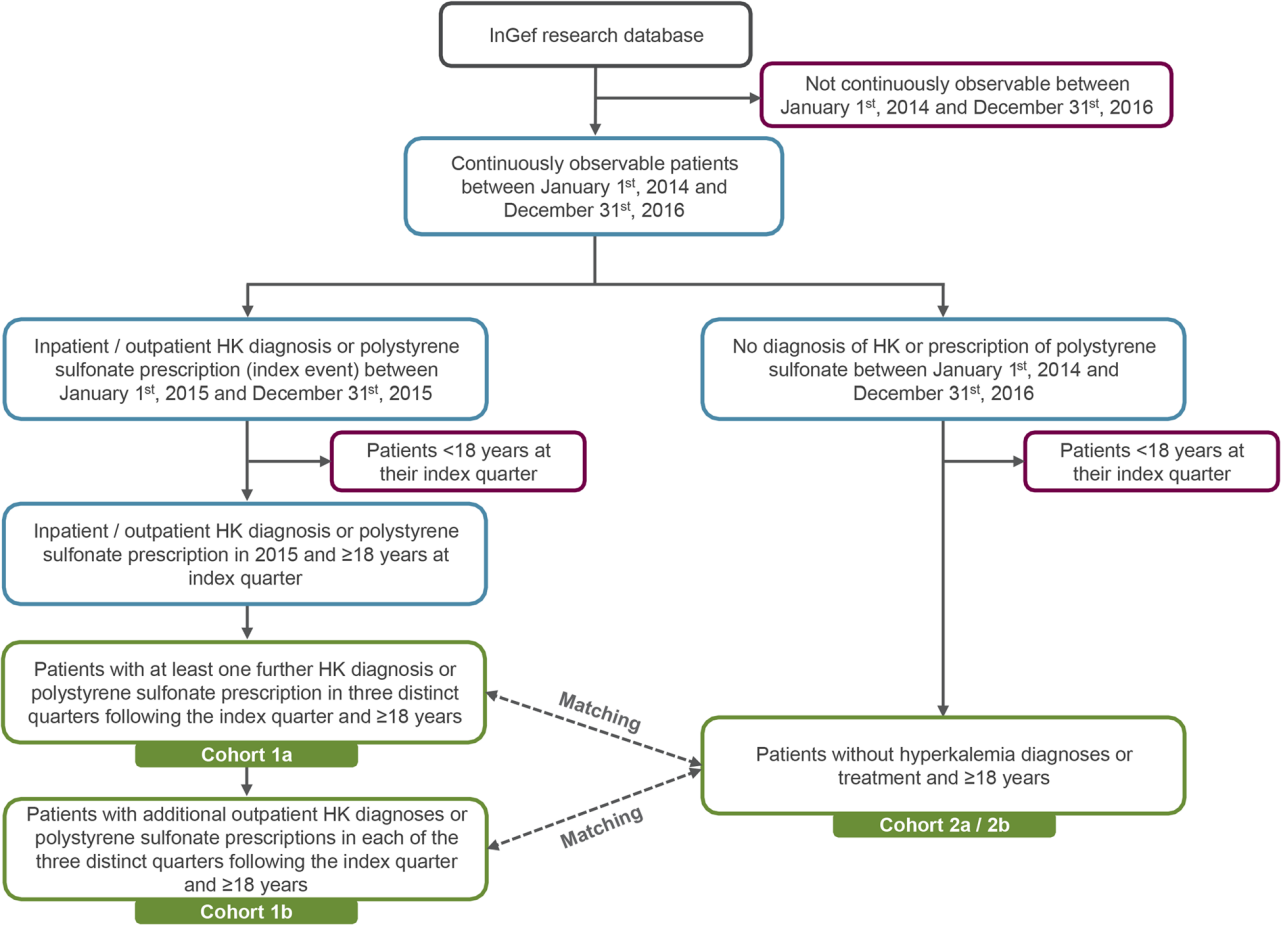
Patient selection steps

Non-acute outpatient treated HK patients and those with chronic HK were identified and defined the main cohorts of interest. To that end, all patients who had at least one inpatient or outpatient ICD-10-GM (International Statistical Classification of Diseases and Related Health Problems, 10th revision, German Modification) diagnosis code of E87.5 “Hyperkalemia” or at least one prescription of polystyrene sulfonate (ATC code: V03AE01), were identified in 2015.. As a next step, we excluded all patients who were < 18 years in the quarter of their index event. Out of these patients, patients with non-acute outpatient treatment for hyperkalemia (cohort 1a) were identified by having at least two HK diagnoses and/or polystyrene sulfonate prescriptions with a restriction to one inpatient diagnosis and a follow-up outpatient diagnosis, indicating non-acute HK treatment.. Out of the cohort of non-acute outpatient treated HK patients, the subgroup of chronic HK patients (cohort 1b) was identified. These patients were required to have outpatient HK diagnoses or polystyrene sulfonate prescriptions in each quarter of the follow-up period. Figure 1 presents an overview of the steps that were performed to identify the respective HK patient cohorts as well as the respective cohort without HK from the database.

### Matching approach

Individuals without hyperkalemia (cohort 2a/2b) were matched 1:1 to each identified hyperkalemia patient of cohort 1a and cohort 1b. Therefore, patients without any diagnosis of HK and without any prescription of polystyrene sulfonate during the whole study period (January 1st, 2014 to December 31st, 2016) were identified from the InGef database (cohort 2a/2b). Index quarters were assigned to adult patients without hyperkalemia based on the distribution of the index quarters in the cohorts 1a and 1b. An exact matching approach without replacement was applied for both matchings of the cohorts 1a and 2a and 1b and 2b, respectively. The matching parameters included age, gender, number of hospitalizations in the index quarter, prescriptions of RAASi in the index quarter (yes/no), and chronic kidney disease with related stages, taking the highest coded stage in the pre-index period into account.

The matching performance was evaluated by calculating absolute standardized differences of the cohorts as well as by statistical testing.

### Outcome assessment

The assessment of patient characteristics included demographics in the index quarter and disease-specific comorbidities, such as CKD, HF, and diabetes mellitus type 2 (T2DM) in the pre-index period. Further, the mean updated Charlson Comorbidity Index (CCI) was assessed for each HK cohort (see Additional file 1).

As the individual number of hospitalizations in the index quarter were taken into account as a matching parameter, the all-cause and HK-related hospitalizations were analyzed in the 3 quarters following the index quarter.

Concomitant treatment was determined for RAASi therapy using ATC codes. Furthermore, use of sodium or calcium polystyrene sulfonate (SPS/CPS) stratified by disease-specific comorbidities within the HK patient cohorts, taking the most severe diagnosis for the respective comorbidity (CKD/HF) into account, was assessed. Prescriptions were analyzed in terms of mean number of prescriptions for SPS/CPS and proportion of heavy users (≥8 prescriptions in total or ≥ 2 prescriptions per quarter) as well as prescribing physician for SPS/CPS in the post-index period.

Healthcare costs stratified by different healthcare sectors, such as inpatient, outpatient, pharmaceuticals, and remedies and aids were assessed in the post-index period.

Time to dialysis initiation was assessed using the Kaplan-Meier method for all HK patients suffering from CKD (all stages) who did not have dialysis in the pre-index period. Therefore, the number of quarters between the first observable HK diagnosis and the occurrence of dialysis in the post index-up period was calculated. Patients were right censored in case of death, sickness fund switch or at the end of the observation period. Dialysis occurrence was assessed by OPS codes and defined EBM codes (see Additional file 1) at four possible timepoints (first quarter, second quarter, third quarter, fourth quarter) to analyze the time to event occurrence within the quarters of the follow-up period. Groups were compared with Log-Rank test.

Study outcomes were analyzed using counts and percentages and compared using the Chi-squared test for all categorical outcomes. Continuous outcomes were analyzed in terms of mean, standard deviation, 0.25 percentile, median, 0.75 percentile, minimum, maximum and sum and compared using the Wilcoxon signed rank test or the paired t test depending on the distribution of the differences.

## Results

### Descriptive analysis

In total, *N* = 3333 patients with non-acute outpatient treated HK (cohort 1a) and *N* = 1693 chronic HK patients (cohort 1b) aged at least 18 years were identified in the database. Outpatient treated HK patients were 70.6 years old on average (SD: 13.3, range: 18–102) and chronic HK patients were 71.2 years old on average (SD: 12.7, range: 21–101). 42.6% of the non-acute outpatient treated HK patients and 43.9% of the chronic HK patients were female.

Almost one third of the patients in both cohorts did not have one of the defined disease-specific comorbidities or any combination thereof. Most non-acute outpatient treated (48.7%) and chronic HK patients (47.5%) had CKD stage 3, 4, or 5 in the pre-index period. Diabetes mellitus type 2 (not sub-classified) had a prevalence of 46.4% in the non-acute outpatient HK cohort and 45.7% in the chronic HK cohort, respectively. The prevalence of heart failure (all grades) alone, in combination with diabetes mellitus type 2 or CKD any stage was 20.4% in non-acute outpatient treated and 18.9% in chronic HK patients (see Table 1).

**Table 1:** Disease-specific comorbidities and CCI of HK patients

Disease (ICD-10-GM code)	Non-acute outpatient treated HK patients ***N*** = 3333		Chronic HK patients ***N*** = 1693	
	n	%	n	%
**CKD**				
CKD stages 1 to 5 (N18.1 + N18.2 + N18.3 + N18.4 + N18.5)	1737	52.1	860	50.8
CKD stage 3 to 5 (N18.3 + N18.4 + N18.5)	1623	48.7	805	47.5
**HF**				
Heart failure (ANY) (I50.11-I50.14, I50.19)	681	20.4	320	18.9
**T2DM**				
Diabetes mellitus, type 2 (E11.-)	1481	46.44	753	44.5
**Comorbidities (CKD, T2DM, HF)**				
**None**	**943**	**28.3**	**483**	**28.5**
**One Comorbidity**	**1203**	**36.1**	**633**	**37.4**
CKD	640	19.2	331	19.6
T2DM	486	14.6	264	15.6
HF	77	2.3	38	2.2
**Two Comorbidities**	**865**	**26.0**	**431**	**25.5**
CKD + T2DM	583	17.5	295	17.4
CKD + HF	192	5.8	88	5.2
T2DM + HF	90	2.7	48	2.8
**Three Comorbidities**	**322**	**9.7**	**146**	**8.6**
**≥ One Comorbidity**	**2390**	**71.7**	**1210**	**71.5**
**Charlson Comorbidity Index (CCI)**				
CCI score: 0	397	11.9	185	10.9
CCI score: 1	502	15.1	283	16.7
CCI score: 2	485	14.6	247	14.6
CCI score: 3	494	14.8	265	15.7
CCI score: ≥4	1455	43.7	713	42.1
Mean CCI score	3.32		3.26	

**Fig. 2:**
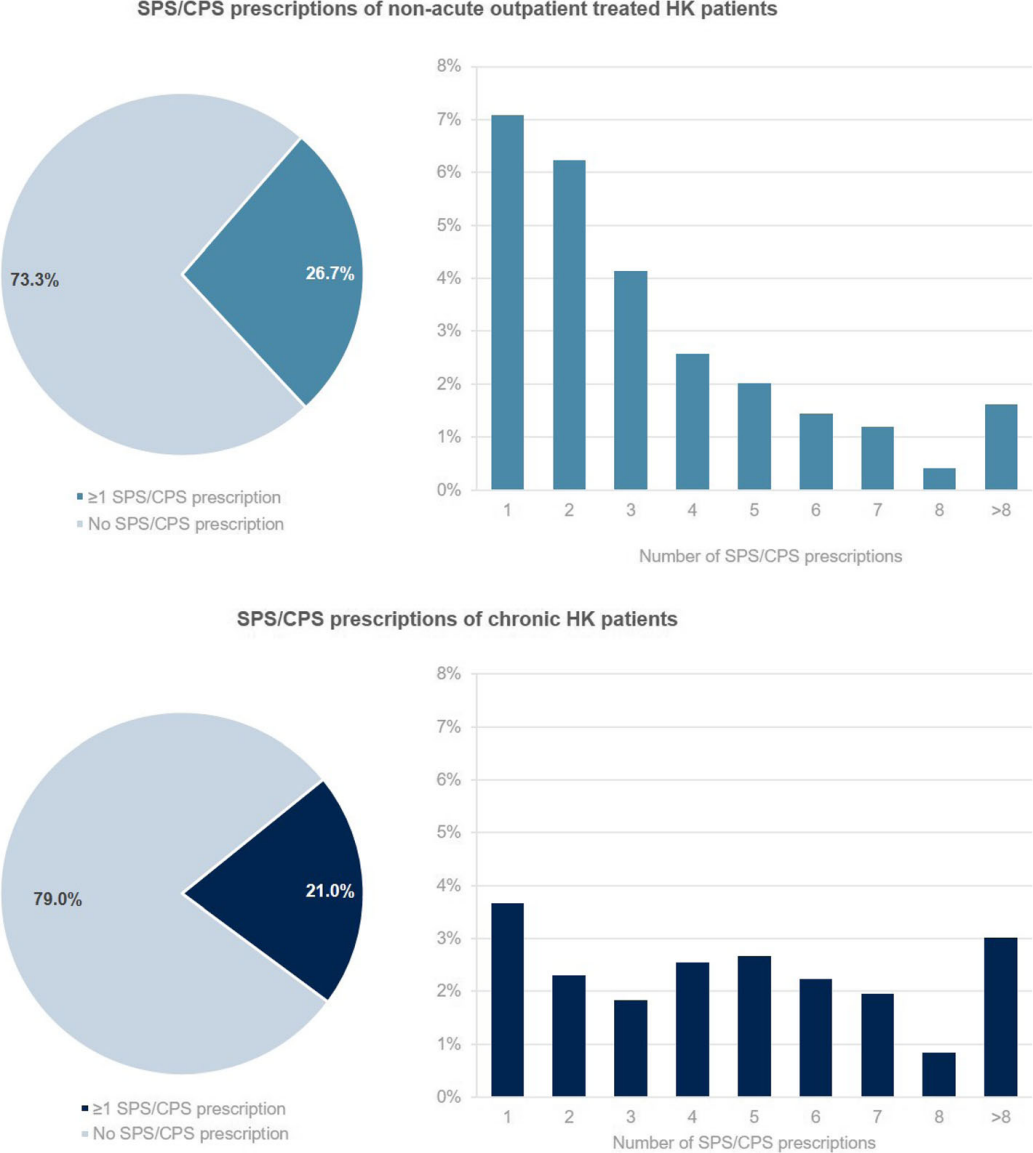
Use of SPS/CPS in patients with non-acute outpatient treated and patients with chronic HK

The analysis of concomitant medication within the individual post-index period of 4 quarters showed that *n* = 1824 (54.7%) of the non-acute outpatient treated HK patients (cohort 1a) and *n* = 927 (54.8%) chronic HK patients (cohort 1b) received at least one prescription of high-ceiling diuretics. Prescriptions for any agents of RAAS inhibitors were present in 1534 (46.0%) and 760 (44.9%) cohorts 1a and 1b, respectively. Patients with at least one prescription of SPS/CPS accounted for *n* = 891 patients (26.7%) in cohort 1a and *n* = 356 patients (21.0%) in cohort 1b. Nephrologists prescribed 47.8% (1a) and 45.7% (1b) of SPS/CPS whereas prescriptions by cardiologists accounted for 0.3% (1a) and 0.3% (1b). The defined heavy use of SPS/CPS was detected in 54 (1.6%) (1a) and 51 (3.0%) (1b) (see Fig. 2).

80.9% of the non-acute outpatient treated HK patients and 79.5% of the chronic HK patients who had at least one SPS/CPS prescription, also incurred a diagnosis of CKD (any stage) in the individual post-index period. A proportion of 35.6% (cohort 1a) and 27.2% (cohort 1b) suffered from CKD stage 5 and received dialysis, indicated by the respective coding and taking the most severe CKD diagnosis in the observation period into account

Moreover, 23.1% of the outpatient treated HK patients as well as 18.5% of the chronic HK patients with at least one SPS/CPS prescription had diagnoses of heart failure with NYHA classes II to IV being the most prevalent NYHA classes (see Table 2).

**Table 2:** Disease-specific comorbidities of patients with and without SPS/CPS use

	Non-acute outpatient treated HK patients (cohort 1a) (***N*** = 3333)				Chronic HK patients (cohort 1b) (***N*** = 1693)			
	Patients with ≥1 SPS/CPS prescription		Patients without SPS/CPS prescription		Patients with ≥1 SPS/CPS prescription		Patients without SPS/CPS prescription	
	n	%	n	%	n	%	n	%
**Chronic kidney disease (most severe diagnosis coding was taken into account)**								
CKD stage 1 & 2	12	1.3	102	4.2	< 5	NA	51	3.8
CKD stage 3	139	15.6	395	16.2	59	16.6	193	14.4
CKD stage 4	198	22.2	245	10.0	102	28.7	134	10.0
CKD stage 5 without dialysis	55	6.2	63	2.6	21	5.9	42	3.1
CKD stage 5 with dialysis	317	35.6	211	8.6	97	27.2	157	11.7
CKD (distinct)	721	80.9	1016	41.6	283	79.5	577	43.2
**Heart failure (most severe diagnosis coding was taken into account)**								
Heart failure NYHA class I	14	1.6	26	1.1	< 5	NA	17	1.3
Heart failure NYHA class II	51	5.7	108	4.4	21	5.9	61	4.6
Heart failure NYHA class III	57	6.4	149	6.1	16	4.5	88	6.6
Heart failure NYHA class IV	63	7.1	136	5.6	19	5.3	64	4.8
Heart failure unspecified	21	2.4	56	2.3	6	1.7	24	1.8
Heart failure (distinct)	206	23.1	475	19.5	66	18.5	254	19.0

### Matching analysis

After matching the non-acute outpatient treated HK patients (1a*) to a non-HK cohort (2a*) and also matching the chronic HK patients (1b*) to a non-HK cohort (2b*), a study population of *n* = 3191 patients with non-acute outpatient treated HK and *n* = 1664 patients with chronic HK with respective control groups remained. After the exact matching, the aforementioned matching parameters were completely balanced with standardized differences of 0 and *p*-values of 1 for all factors (see Additional file 1) for the matching parameters before and after matching]. The mean number of hospitalizations in the index quarter was 0.5 (cohorts 1a*/2a*) and 0.4 (cohorts 1b*/2b*), respectively. Furthermore, half of the patients of all matched cohorts (50.0% vs. 50.1%) did not have a CKD diagnosis and about 18% of the patients had a diagnosis of CKD stage 5 (see Supplementary Material for more detailed information).

Both HK cohorts showed a higher number of hospitalizations compared to non-HK patients (cohorts a*: 2732 vs. 1749/cohorts b*: 1125 vs. 930). HK patients had on average more hospitalizations than patients in the control group (cohorts a*: 0.9 vs. 0.5, *p* ≤ 0.001/cohorts b*: 0.7 vs. 0.6, *p* = 0.002). HK-related hospitalizations with HK as main diagnosis accounted for around 1% of the identified inpatient stays of HK patients.

The total number of dialysis events in the post-index period was highest in cohort 1a* with 8324 events (3132 cohort 2a*), whereas cohort 1b* had 4602 events (1509 cohort 2b*). The assessment of dialysis initiation in the one-year follow up revealed that individuals with HK had a twofold higher initiation rate than their matched counterparts. Further, the time to dialysis initiation was assessed with Kaplan-Meier (see Additional file 1 for the graphs), taking the occurrence of dialysis in each quarter into account. Individuals without hyperkalemia in the respective cohorts had a higher probability of initiating dialysis treatment in the first four quarters than their matched hyperkalemia counterparts. Accordingly, the average time until dialysis initiation was higher for both hyperkalemia cohorts (cohort 1a* mean in quarters 1.3 vs. cohort 2a* mean in quarters 1.0; and cohort 1b* mean in quarters 1.0 vs. cohort 2b* mean in quarters 0.8;) (see Table 3).

**Table 3:** Time-to-dialysis initiation event

	No. of patients with dialysis initiation event		Average length of observation time until dialysis initiation event (in quarters)				
	n	%	Mean	STD	Median	Min	Max
Patients with outpatient hyperkalemia (cohort 1a*)	118	10.5	1.3	1.2	1.0	0.0	3.0
Patients without hyperkalemia (cohort 2a*)	62	4.8	1.0	1.1	1.0	0.0	3.0
Patients with chronic hyperkalemia (cohort 1b*)	51	8.7	1.0	1.2	1.0	0.0	3.0
Patients without hyperkalemia (cohort 2b*)	28	4.2	0.8	1.0	0.0	0.0	3.0

Note: *refers to the matched cohorts and their respective counterparts

**Fig. 3:**
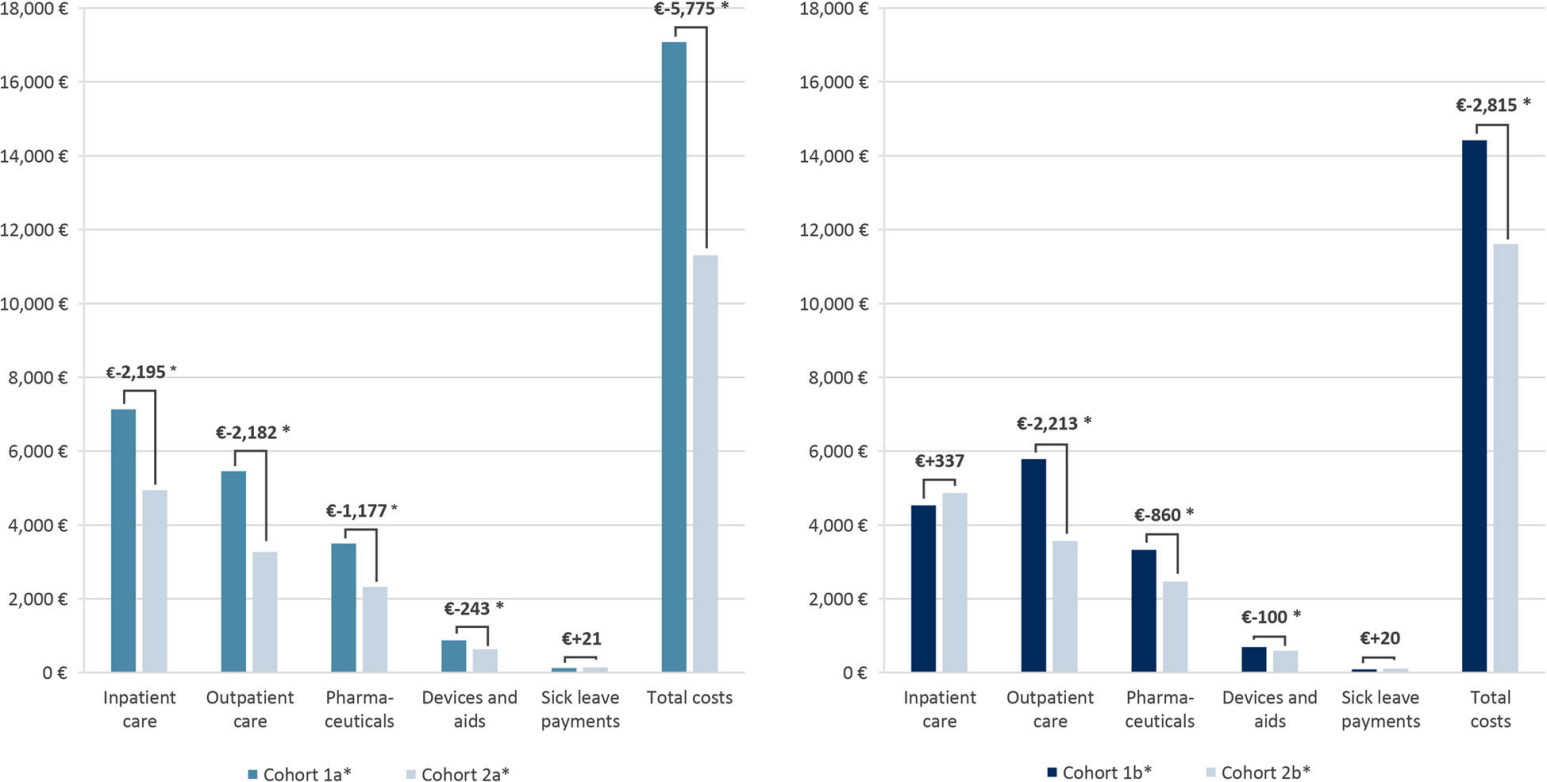
Mean healthcare costs of the matched HK and non-HK cohorts

The mean total healthcare costs in the post-index period of 1 year (4 quarters) were significantly higher for both HK cohorts and their matched counterparts (cohorts a*: €17,076 vs. €11,301, *p* ≤ 0.001/cohorts b*: €14,421 vs. €11,606, *p* ≤ 0.001). Additionally, when comparing cohort 1a* and 2a*, significant differences in mean costs referring to the inpatient and outpatient care setting, as well as pharmaceuticals and devices and aids, could be reported. The observed higher costs in the chronic HK cohort 1b* were not significant for inpatient care costs and sick leave payment. It can be noted that the assessed population was on average over 70 years of age, therefore the sick leave costs were quite low. Significantly higher costs were observed in outpatient care, pharmaceuticals and devices and aids with differences in the mean costs (see Fig. 3). The costs of HK-related hospitalizations for inpatient care accounted for 29.2 and 20.7% for cohort 1a* and 1b* respectively.

## Discussion

To our knowledge, this is the first retrospective claims data analysis assessing the burden and healthcare costs of non-acute outpatient treated HK and chronic HK in the German healthcare setting. The InGef research database utilized for this study is adjusted to represent the German population in terms of age and gender and further shows representativeness regarding hospitalizations, mortality rate, and prescriptions which was validated by a recent study [16]. Health insurance in Germany is statutory and covers almost 85% of the population and the contents of reimbursement are set in social law, offering the same comprehensive benefit package across individual statutory health insurances [17]. Therefore, the study results can be considered reliable in the context of German SHI real world evidence.

It is often stated that hyperkalemia is most commonly associated with diseases such as CKD, DM or HF. But little is known about the prevalence of HK in general or as a comorbidity of one of the aforementioned diseases. A Swedish observational study revealed an incidence of hyperkalemia of 7% within adult individuals [2]. Another study published by Einhorn and colleagues [13] compared the occurrence of hyperkalemia between patients with and without CKD in veteran patients with at least one hospitalization and one potassium measurement during a 1 year time period. The prevalence of HK in patients with any stage of CKD was 26.6% whereas only 8.9% of patients without chronic kidney disease showed potassium values of ≥5.5 mEq/L. In the same study, the proportion of patients with hyperkalemia for the whole study population (13.7%), patients with diabetes (19.5%) and who received ACE inhibitors and/or ARB (16.9%) where stated.

In this analysis, HK was the primary selection criterion to identify patients with non-acute outpatient treated or chronic HK and comorbidities were assessed for these cohorts in a second step. It was shown that more than 70% of patients in both cohorts suffered from at least one condition of CKD, HF or T2DM and that the proportions were higher in patients receiving at least one prescription of SPS/CPS especially for CKD. The use of this HK-specific medication was rather low with 26.6% of patients with at least one prescription in cohort 1a and 20.9% in cohort 1b.

Furthermore, the results of the analysis showed that the number of patients with dialysis initiation was significantly higher in both HK cohorts than in the non-HK cohorts. Non-acute outpatient treated HK patients (cohort 1a) had the highest total number of dialysis events, while the mean number of dialysis events was highest in patients with chronic HK (cohort 1b) who had an average number of 90 dialysis events in the individual post-index period. Taking these results into account, HK is associated with a higher risk of dialysis initiation and the group of patients suffering from chronic HK have even more dialysis events than the non-acute outpatient treated HK patients. However, it should be noted, that individuals were censored upon the occurrence of death, therefore, individuals not deceasing might have had a longer time period under risk. An Italian prospective study showed that, in addition, the likelihood for end stage renal disease (ESRD) and, therefore, the need for dialysis increases with a higher level of serum potassium [18].

Hyperkalemia was determined as a predictor of total costs in patients receiving RAASi treatment for all disease cohorts of chronic kidney disease, heart failure and diabetes by Epstein et al. [19]. For patients with chronic kidney disease and heart failure it was recently shown by Polson and colleagues [20], that hyperkalemia is linked to higher medical costs in patients with and without RAASi treatment. In an analysis by Betts and colleagues [11] for HK in the US the average all-cause costs added up to $15,983. The results of this analysis are in line with the aforementioned publications and show that especially non-acute outpatient treated hyperkalemia patients show significantly higher total healthcare costs and that hyperkalemia related hospitalizations account for more than 20% of inpatient care costs in both cohorts.

### Limitations

In general, claims data are considered an appropriate source to explore and analyze health-relevant real world evidence queries related to, but not limited to, epidemiology and burden of illness, as this data is recorded for reimbursement purposes and not connected to the purpose of the respective study, offering the possibility of less selection bias. Nevertheless, there are limitations related to the use of claims data which need to be considered when interpreting the study results. Firstly, claims data are collected for the purpose of accounting and unless data linkage is performed, no clinical data is available. In this context, the unavailability of acid-base status or the adoption of a low potassium diet should be taken into account when interpreting the results, as the definition of hyperkalemia is more nuanced and cannot be fully addressed without additional clinical information. Further, the results should be considered with the background of the anticipated coding quality and the need for the respective individuals to seek treatment. Additionally, diagnoses in the outpatient setting are coded on a quarterly basis and cannot be attributed to an exact date. The private sector was not covered by this analysis, as the data pool only includes sickness funds from the SHI system. Concerning the performed matching, unobserved variables may be unequally distributed in the groups, which might lead to potentially biased results.

## Conclusions

To date, hyperkalemia prevalence has been assessed by analyzing overall comorbid conditions, such as CKD and DM, and reporting the comorbid occurrence of hyperkalemia. Within this study, the prevalence of hyperkalemia was the main focus irrespective of any comorbid diseases. However, our analysis revealed that HK patients are multi-morbid and present with an increased prevalence of CKD and DM, which has previously been shown in other analyses.

Given the increased disease burden and the associated healthcare costs of patients with non-acute outpatient treated or chronic HK, in comparison to their matched counterparts, a necessity for treatment improvement could be suggested. On the other hand, the use of the only HK-specific medication (SPS / CPS) that was available during the study period, was rather low.

A possible conclusion is that an adequate treatment of HK could reduce the patients’ hospitalizations, the need for dialysis and in turn the healthcare costs from a long term perspective, which warrants further research.

## Supplementary information

**Additional file 1.** Matching parameters before and after matching. Description of data: Matching variables included in the exact 1:1 matching for non-acute outpatient HK vs. non-HK patients and for acute HK vs. non-HK patients. Coding lists including dialysis, hyperkalemia, RAASi, CKD stages, heart failure, and diabetes mellitus identification. Charlson Comorbidity Index overview. Time to dialysis initiation (Kaplan Meier curves).

## Data Availability

All data generated or analyzed during this study are included in this published article [and its supplementary information files]. The data that support the findings of this study are not publicly available as they are owned by the statutory health insurances. Data requests need to be granted by the InGef and the participating health insurances.

## Abbreviations

ACE: Angiotensin-converting enzyme
ARB: Angiotensin receptor blockers
ATC: Anatomical therapeutic chemical classification system
CKD: Chronic kidney disease
CPS: Calcium polystyrene sulfonate
CVD: Cardiovascular disease
DESTATIS: Statistisches Bundesamt (Federal Office of Statistics)
DM: Diabetes mellitus
EBM: Einheitlicher Bewertungsmassstab (Practitioners’ Fee Scale within the Statutory Health Insurance Scheme)
ESRD: End stage renal disease
GFR: Glomerular filtration rate
HF: Heart failure
HK: Hyperkalemia
ICD-10-GM: International Statistical Classification of Diseases and Related Health Problems, 10th revision, German Modification
InGef: “Institut fuer angewandte Gesundheitsforschung Berlin” (Institute for applied health research, Berlin)
NYHA: New York Heart Association
OPS: Operationen- und Prozedurenschluessel (Key of operations and procedures)
Q: Quarter
RAASi: Renin-angiotensin-aldosterone system inhibitors
SD: Standard deviation
SHI: Statutory Health System
SPS: Sodium polystyrene sulfonate
T2DM: Type 2 diabetes mellitus
