# Supplementary material for: The burden of hyperkalemia in Germany – a real world evidence study assessing the treatment and costs of hyperkalemia
Source: BMC Nephrol. 2020 Aug 8;21:332. doi: 10.1186/s12882-020-01942-2 (PMC7414716; doi:10.1186/s12882-020-01942-2)

Table A-1: Matching parameters before matching

|  | **Cohort 1a (N=3,333)** | | | | | | | **Cohort 2a (N=2,915,312)** | | | | | | | **p value** |
| --- | --- | --- | --- | --- | --- | --- | --- | --- | --- | --- | --- | --- | --- | --- | --- |
|  | **n** | **%** | **Mean** | **Std** | **Median** | **Min** | **Max** | **n** | **%** | **Mean** | **Std** | **Median** | **Min** | **Max** |  |
| Age |  |  | 70.6 | 13.3 | 73 | 18 | 102 |  |  | 52.0 | 18.2 | 52 | 18 | 108 | 0.000 |
| Gender (Female) | 1,419 | 42.6 |  |  |  |  |  | 1,477,545 | 50.7 |  |  |  |  |  | 0.000 |
| No. of hospi-talizations in index quarter | 1,898 |  | 0.6 | 1.0 | 0 | 0 | 7 | 296,585 |  | 0.1 | 0.4 | 0 | 0 | 23 | 0.000 |
| RAASI treatment in index quarter | 1,123 | 33.7 |  |  |  |  |  | 362,201 | 12.4 |  |  |  |  |  | 0.000 |
| No CKD diagnosis | 1,596 | 47.9 |  |  |  |  |  | 2,837,779 | 97.3 |  |  |  |  |  | 0.000 |
| CKD stage 1 | 24 | 0.7 |  |  |  |  |  | 6,605 | 0.2 |  |  |  |  |  | 0.000 |
| CKS stage 2 | 90 | 2.7 |  |  |  |  |  | 20,282 | 0.7 |  |  |  |  |  | 0.000 |
| CKD stage 3 | 534 | 16.0 |  |  |  |  |  | 39,340 | 1.3 |  |  |  |  |  | 0.000 |
| CKD stage 4 | 443 | 13.3 |  |  |  |  |  | 7,884 | 0.3 |  |  |  |  |  | 0.000 |
| CKD stage 5 | 646 | 19.4 |  |  |  |  |  | 3,422 | 0.1 |  |  |  |  |  | 0.000 |
|  | **Cohort 1b (N=1,693)** | | | | | | | **Cohort 2b (N=2,913,115)** | | | | | | | **p value** |
|  | **n** | **%** | **Mean** | **Std** | **Median** | **Min** | **Max** | **n** | **%** | **Mean** | **Std** | **Median** | **Min** | **Max** |  |
| Age |  |  | 70.6 | 13.3 | 73 | 18 | 102 |  |  | 52.0 | 18.2 | 52 | 18 | 108 | 0.000 |
| Gender (Female) | 1,419 | 42.6 |  |  |  |  |  | 1,477,545 | 50.7 |  |  |  |  |  | 0.000 |
| No. of hospi-talizations in index quarter | 1,898 |  | 0.6 | 1.0 | 0 | 0 | 7 | 296,585 |  | 0.1 | 0.4 | 0 | 0 | 23 | 0.000 |
| RAASI treatment in index quarter | 1,123 | 33.7 |  |  |  |  |  | 362,201 | 12.4 |  |  |  |  |  | 0.000 |
| No CKD diagnosis | 1,596 | 47.9 |  |  |  |  |  | 2,837,779 | 97.3 |  |  |  |  |  | 0.000 |
| CKD stage 1 | 24 | 0.7 |  |  |  |  |  | 6,605 | 0.2 |  |  |  |  |  | 0.000 |
| CKS stage 2 | 90 | 2.7 |  |  |  |  |  | 20,282 | 0.7 |  |  |  |  |  | 0.000 |
| CKD stage 3 | 534 | 16.0 |  |  |  |  |  | 39,340 | 1.3 |  |  |  |  |  | 0.000 |
| CKD stage 4 | 443 | 13.3 |  |  |  |  |  | 7,884 | 0.3 |  |  |  |  |  | 0.000 |
| CKD stage 5 | 646 | 19.4 |  |  |  |  |  | 3,422 | 0.1 |  |  |  |  |  | 0.000 |

Table A-2: Matching parameters after matching

|  | **Cohort 1a* (N=3,191)** | | | | | | | **Cohort 2a* (N=3,191)** | | | | | | | **p value** |
| --- | --- | --- | --- | --- | --- | --- | --- | --- | --- | --- | --- | --- | --- | --- | --- |
|  | **n** | **%** | **Mean** | **Std** | **Median** | **Min** | **Max** | **n** | **%** | **Mean** | **Std** | **Median** | **Min** | **Max** |  |
| Age |  |  | 71.1 | 12.9 | 74 | 18 | 102 |  |  | 71.1 | 12.9 | 74 | 18 | 102 | 1.000 |
| Gender (Female) | 1,373 | 43.0 |  |  |  |  |  | 1,373 | 43.0 |  |  |  |  |  | 1.000 |
| No. of hospi-talizations in index quarter | 1,554 |  | 0.5 | 0.9 | 0 | 0 | 7 | 1,554 |  | 0.5 | 0.9 | 0 | 0 | 7 | 1.000 |
| RAASI treatment in index quarter | 1,061 | 33.2 |  |  |  |  |  | 1,061 | 33.2 |  |  |  |  |  | 1.000 |
| No CKD diagnosis | 1,596 | 50.0 |  |  |  |  |  | 1,596 | 50.0 |  |  |  |  |  | 1.000 |
| CKD stage 1 | 21 | 0.7 |  |  |  |  |  | 21 | 0.7 |  |  |  |  |  | 1.000 |
| CKS stage 2 | 87 | 2.7 |  |  |  |  |  | 87 | 2.7 |  |  |  |  |  | 1.000 |
| CKD stage 3 | 521 | 16.3 |  |  |  |  |  | 521 | 16.3 |  |  |  |  |  | 1.000 |
| CKD stage 4 | 407 | 12.8 |  |  |  |  |  | 407 | 12.8 |  |  |  |  |  | 1.000 |
| CKD stage 5 | 559 | 17.5 |  |  |  |  |  | 559 | 17.5 |  |  |  |  |  | 1.000 |
|  | **Cohort 1b* (N=1,664)** | | | | | | | **Cohort 2b* (N=1,664)** | | | | | | | **p value** |
|  | **n** | **%** | **Mean** | **Std** | **Median** | **Min** | **Max** | **n** | **%** | **Mean** | **Std** | **Median** | **Min** | **Max** |  |
| Age |  |  | 71.5 | 12.4 | 74 | 23 | 101 |  |  | 71.5 | 12.4 | 74 | 23 | 101 | 1.000 |
| Gender (Female) | 735 | 44.2 |  |  |  |  |  | 735 | 44.2 |  |  |  |  |  | 1.000 |
| No. of hospi-talizations in index quarter | 323 |  | 0.4 | 0.7 | 0 | 0 | 6 | 323 |  | 0.4 | 0.7 | 0 | 0 | 6 | 1.000 |
| RAASI treatment in index quarter | 531 | 31.9 |  |  |  |  |  | 531 | 31.9 |  |  |  |  |  | 1.000 |
| No CKD diagnosis | 833 | 50.1 |  |  |  |  |  | 833 | 50.1 |  |  |  |  |  | 1.000 |
| CKD stage 1 | 10 | 0.6 |  |  |  |  |  | 10 | 0.6 |  |  |  |  |  | 1.000 |
| CKS stage 2 | 43 | 2.6 |  |  |  |  |  | 43 | 2.6 |  |  |  |  |  | 1.000 |
| CKD stage 3 | 250 | 15.0 |  |  |  |  |  | 250 | 15.0 |  |  |  |  |  | 1.000 |
| CKD stage 4 | 227 | 13.6 |  |  |  |  |  | 227 | 13.6 |  |  |  |  |  | 1.000 |
| CKD stage 5 | 301 | 18.1 |  |  |  |  |  | 301 | 18.1 |  |  |  |  |  | 1.000 |

Table A-3: Dialysis Identification

| **OPS codes** | **Description** |
| --- | --- |
| 8-854* | Hemodialysis |
| 8-855* | Hemodiafiltration |
| 8-856 | Hemoperfusion |
| 8-857* | Peritoneal dialysis |
| 8-85a | Dialysis caused to insufficient functional imaging and failure of a renal transplant |
| **EBM codes** | **Description** |
| 04562 | Additional charge for the continuous care of a dialysis patient (pediatric patients) |
| 04564 | Additional charge for pediatric nephrological care for patients with hemodialysis as center or practice dialysis, home dialysis or centralized home dialysis, or intermittent peritoneal dialysis |
| 04565 | Additional charge for pediatric nephrological care during a peritoneal dialysis |
| 04566 | Surcharge for GOP 04564 and 04565 for implementing a training dialysis (for pediatric patients) |
| 13602 | Additional charge for the continuous care of a dialysis patient |
| 13610 | Additional charge for medical care for patients with hemodialysis as center or practice dialysis, home dialysis or centralized home dialysis, or intermittent peritoneal dialysis |
| 13611 | Additional charge for medical care during a peritoneal dialysis |
| 13612 | Surcharge for GOP 13610 or 13611 for implementing a training dialysis |
| 40815 | Lump sum for material expenses for the implementation of hemodialysis incl. special procedures for patients ≤ 18 years |
| 40816 | Lump sum for material expenses for the implementation of peritoneal dialysis incl. special procedures for patients ≤ 18 years |
| 40817 | Lump sum for material expenses for the implementation of peritoneal dialysis incl. special procedures for patients ≤ 18 years (for dialysis at the place of residence) |
| 40818 | Lump sum for material expenses for the implementation of hemodialysis incl. special procedures for patients ≤ 18 years (for dialysis during holiday) |
| 40819 | Lump sum for material expenses for the implementation of peritoneal dialysis for patients ≤ 18 years (for dialysis during holiday) |
| 40823 | Lump sum for material expenses for the implementation of hemodialysis as center or practice dialysis, or centralized home dialysis, incl special procedures for patients > 18 years |
| 40824 | Lump sum for material expenses for the implementation of hemodialysis as center or practice dialysis, or centralized home dialysis, incl special procedures for patients > 18 years (for dialysis at the place of residence) |
| 40825 | Lump sum for material expenses for the implementation of peritoneal dialysis, or home hemodialysis for patients > 18 years |
| 40826 | Lump sum for material expenses for the implementation of peritoneal dialysis for patients > 18 years (for dialysis at the place of residence) |
| 40827 | Lump sum for material expenses for the implementation of intermittent peritoneal dialysis, or home hemodialysis for patients > 18 years (for dialysis at the place of residence) |
| 40828 | Lump sum for material expenses for the implementation of hemodialysis or peritoneal dialysis as center or practice dialysis, home dialysis, or centralized home dialysis incl. special procedures for patients > 18 years (for dialysis during holiday) |
| 40829 | Surcharge for the lump sum 40823 or 40825 for patients  60 – 69 years |
| 40830 | Surcharge for the lump sum 40824, 40826, or 40827 for patients 60 – 69 years |
| 40831 | Surcharge for the lump sum 40823 or 40825 for patients  70 – 79 years |
| 40832 | Surcharge for the lump sum 40824, 40826, or 40827 for patients 70 – 79 years |
| 40833 | Surcharge for the lump sum 40823 or 40825 for patients  ≥ 80 years |
| 40834 | Surcharge for the lump sum 40824, 40826, or 40827 for patients ≥ 80 years |
| 40837 | Surcharge for the lump sum 40816 or 40825 for the intermittent peritoneal dialysis |
| 40838 | Surcharge for the lump sum 40817, 40819, 40827, or 40828 for the intermittent peritoneal dialysis |

Table A-4: List of Codes – Identification of Hyperkalemia

| **ICD-10-GM Codes** | **Description** |
| --- | --- |
| E87.5 | Hyperkalemia |
| **ATC Codes** | **Description** |
| V03AE01 | Polystyrene sulfonate |

Table A-5: List of Codes – Presence of RAASi treatment

| **ATC Codes** | **Description** |
| --- | --- |
| C03AA* | ACE inhibitors |
| C09BA* | ACE inhibitors and diuretics |
| C09BB* | ACE inhibitors and calcium inhibitors |
| C09BX01 | ACE inhibitors and other combinations |
| C09CA* | ARB |
| C09DA* | Angiotensin II antagonists and diuretics, |
| C09DB* | Angiotensin II antagonists and calcium channel blockers |
| C03DA* | Aldosterone-antagonists |
| C03EC* | Aldosterone-antagonists and low-ceiling diuretics |
| C03ED* | Aldosterone-antagonists and high-ceiling diuretics |
| C09XA* | Renin inhibitors |

Table A-6: List of Codes – CKD Stages

| **ICD-10-GM Codes** | **Description** |
| --- | --- |
| N18.1 | Chronic kidney disease, stage 1 |
| N18.2 | Chronic kidney disease, stage 2 |
| N18.3 | Chronic kidney disease, stage 3 |
| N18.4 | Chronic kidney disease, stage 4 |
| N18.5 | Chronic kidney disease, stage 5 |

Table A-7: List of Codes – Heart Failure

| **ICD-10-GM Codes** | **Description** |
| --- | --- |
| I50.11 | Heart failure, NYHA class 1 |
| I50.12 | Heart failure, NYHA class 2 |
| I50.13 | Heart failure, NYHA class 3 |
| I50.14 | Heart failure, NYHA class 4 |
| I50.19 | Heart failure, not specified |

Table A-8: List of Codes – Diabetes mellitus

| **ICD-10-GM Codes** | **Description** |
| --- | --- |
| E11.- | Diabetes mellitus, type 2 |

Table A-9: Charlson Comorbidity Index

| **ICD-10-GM code** | **Comorbid Condition** | **Charlson Weight^a^** | **Updated Charlson Weight^b^** |
| --- | --- | --- | --- |
| I21, I22, I23, I252, I258 | Myocardial infarction | 1 | 0 |
| I099, I110, I130, I132, I255, I420, I425, I426, I427, I428, I429, I43, I50, P290 | Congestive heart failure | 1 | 2 |
| I70, I71, I731, I738, I739, I771, I790, I792, K551, K558, K559, Z958, Z959 | Peripheral vascular disease | 1 | 0 |
| G45, G450, G451, G452, G454, G458, G459, G46, I60, I61, I62, I63, I64, I65, I66, I67, I68, I69 | Cerebrovascular disease | 1 | 0 |
| F00, F01, F02, F03, F051, G30, G311 | Dementia | 1 | 2 |
| I278, I279, J40, J41, J42, J43, J44, J45, J46, J47, J60, J61, J62, J63, J64, J65, J67, J684, J701, J703 | Pulmonary disease | 1 | 1 |
| M05, M06, M060, M063, M069, M315, M32, M33, M332, M34, M351, M353, M360 | Connective tissue disorder | 1 | 1 |
| K25, K26, K27, K28 | Peptic ulcer disease | 1 | 0 |
| B18, K700, K701, K702, K703, K709, K713, K714, K715, K717, K73, K74, K760, K762, K763, K764, K768, K769, Z944 | Mild liver disease | 1 | 2 |
| E100, E101, E106, E108, E109, E110, E111, E116, E118, E119, E120, E121, E126, E128, E129, E130, E131, E136, E138, E139, E140, E141, E146, E148, E149 | Diabetes without chronic complication | 1 | 0 |
| E102, E103, E104, E105, E107, E112, E113, E114, E115, E117, E122, E123, E124, E125, E127, E132, E133, E134, E135, E137, E142, E143, E144, E145, E147 | Diabetes with chronic complication | 2 | 1 |
| G041, G114, G801, G802, G81, G82, G820, G821, G822, G830, G831, G832, G833, G834, G839 | Paraplegia | 2 | 2 |
| I12, I120, I13, I131, N01, N03, N032, N033, N035, N036, N037, N052, N053, N054, N055, N056, N057, N072, N073, N074, N18, N19, N25, N250, Z490, Z491, Z492, Z940, Z992 | Renal disease | 2 | 1 |
| C00, C01, C02 , C03, C04, C05, C06, C07, C08, C09, C10, C11, C12, C13, C14, C15, C16, C17, C18, C19, C20, C21, C22, C23, C24, C25, C26, C30, C31, C32, C33, C34, C37, C38, C39, C40, C41, C43, C44, C45, C46, C47, C48, C49, C50, C51, C52, C53, C54, C55, C56, C57, C58, C60, C61, C62, C63, C64, C65, C66, C67, C68, C69, C70, C71, C72, C73, C74, C75, C76, C80, C81, C82, C83, C84, C85, C88, C90, C91, C92, C93, C94, C95, C96, C97 | Any malignancy, including lymphoma and leukemia, except malignant neoplasm of skin | 2 | 2 |
| I850, I859, I864, I982, K704, K711, K721, K729, K765, K766, K767 | Severe liver disease | 3 | 4 |
| C77, C78, C79 | Metastatic cancer | 6 | 6 |
| B20, B21, B22, B23, B24 | HIV | 6 | 4 |

Time-to dialysis initiation – Kaplan-Meier

Group 1a*


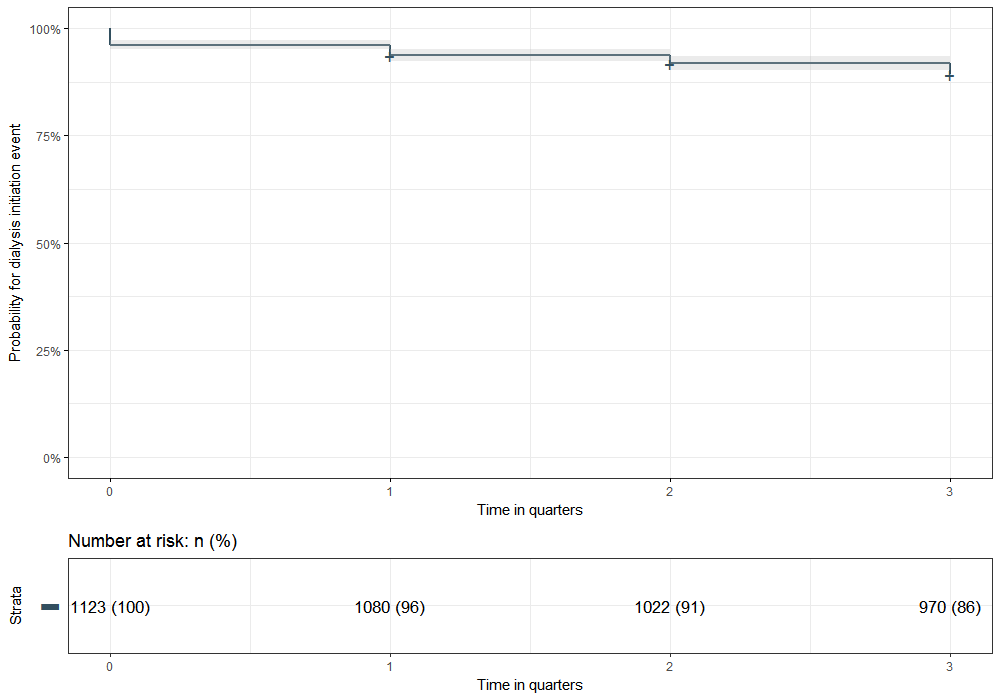


Group 2a*


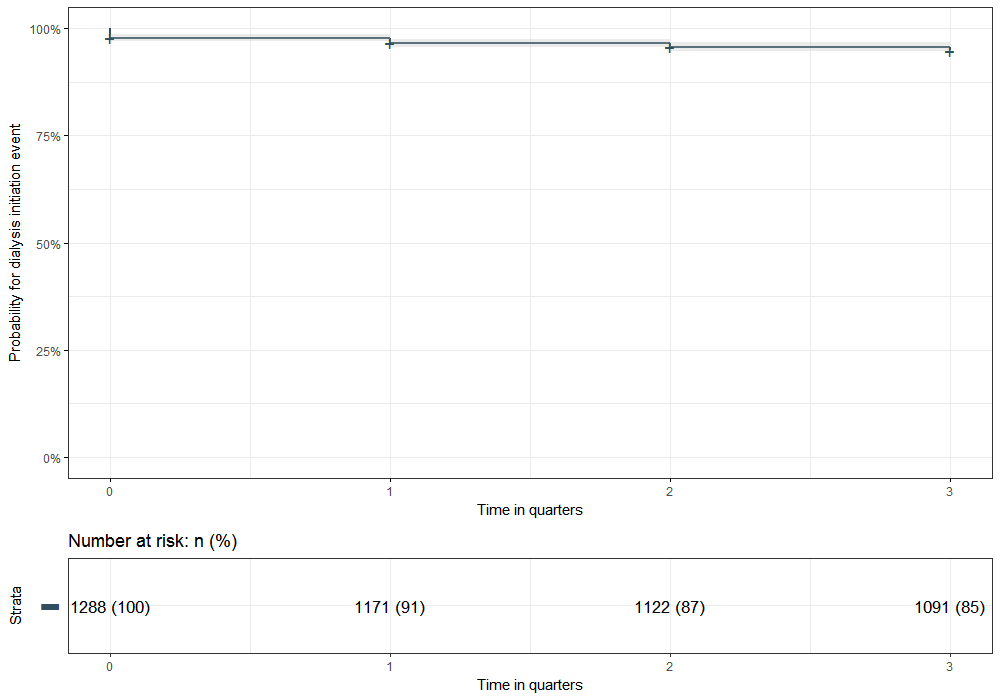


Group 1b*


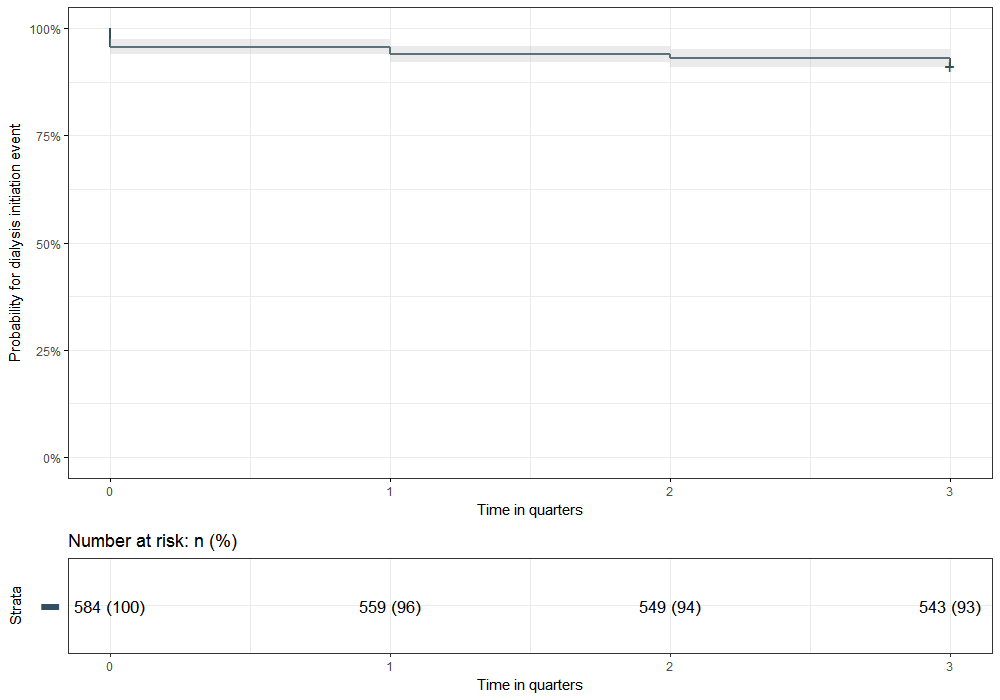


Group 2b*


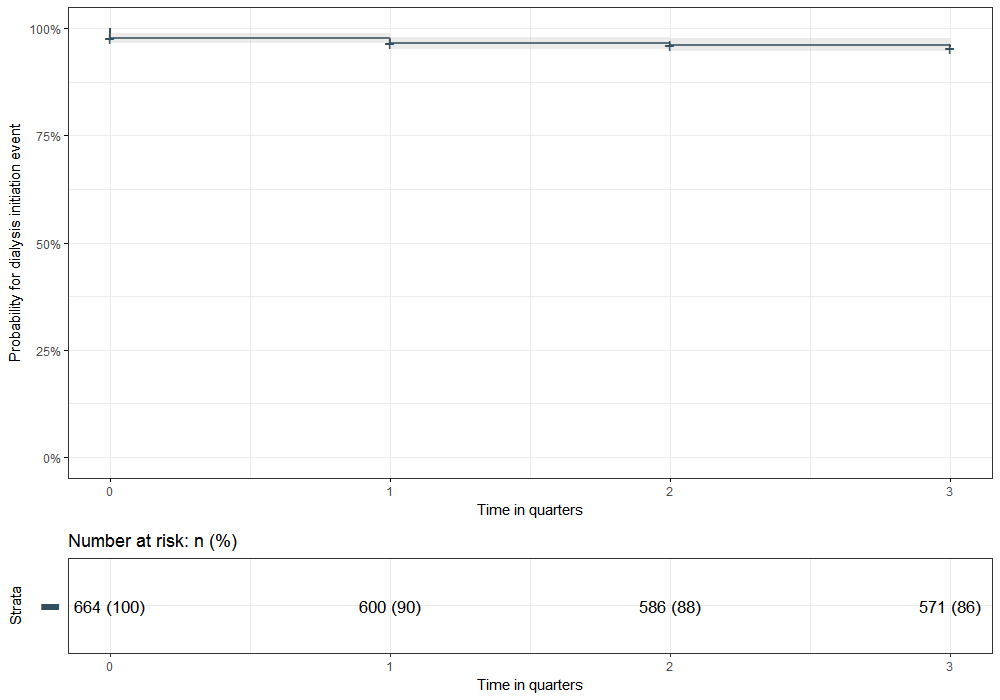

Supplement: Supplementary file 1 — Additional file 1. Matching parameters before and after matching. Description of data: Matching variables included in the exact 1:1 matching for non-acute outpatient HK vs. non-HK patients and for acute HK vs. non-HK patients. Coding lists including dialysis, hyperkalemia, RAASi, CKD stages, heart failure, and diabetes mellitus identification. Charlson Comorbidity Index overview. Time to dialysis initiation (Kaplan Meier curves). [file 12882_2020_1942_MOESM1_ESM.docx]
